# Supplementary material for: Sensorimotor synchronization to music reduces pain
Source: PLoS One. 2023 Jul 28;18(7):e0289302. doi: 10.1371/journal.pone.0289302 (PMC10381080; doi:10.1371/journal.pone.0289302)
Supplement: S1 Protocol — (DOCX) [file pone.0289302.s001.docx]

**Forskningsprotokoll**

# Tittel/navn på prosjekt

Reducing pain thresholds using a focused music listening technique

# Bakgrunn

Et av de mest etablerte funnene innenfor bruk av musikk innen medisin er reduksjon av smerte ved bruk av musikk. Meta-analyser antyder derimot at smertereduksjons-effekten til musikk er små, til tross for dens høye reliabilitet. Musikkens kliniske relevans er derfor ikke godt etablert.

# Problemstilling

Tidligere studier som har undersøkt smertereduksjonseffekten som musikk kan medføre, har dog bare benyttet seg av paradigmer innenfor passiv lytting til musikk. Teknikker hvor en benytter seg av en mer aktiv lytteteknikk har trolig større smertereduksjonseffekt. Dette prosjektet vil undersøke dette problemet, ved å sammenligne smertereduseringseffekten ved bruk av passiv lytting og aktiv lytting.

# Formål

Et positivt utfall kan ha signifikant påvirkning på hvordan bruk av musikk under medisinske prosedyrer kan lindre smerte og redusere psykologiske symptomer knyttet til smerte, som blant annet angst og bekymring.

# METODE

**Design**

Studien er et eksperimentelt repetert within-subject målingsdesign som innebærer at forsøkspersonene blir påført et ubehag ved bruk av Wagners pressalgometer Force One.

Plan Passiv-Aktiv

- Passiv lytting først
- Markerer finger som skal trykkes på
- Gir instrukser
- Gjennomfører trykking fra 0 til 2kg i løpet av 10 sekunder
- Spørreskjema
- Aktiv lytting
- Gir instrukser
- Gjennomfører trykking fra 0 til 2kg i løpet av 10 sekunder Spørreskjema

Plan Aktiv-Passiv

- Aktiv lytting først
- Markerer finger som skal trykkes på
- Gir instrukser
- Gjennomfører trykking fra 0 til 2kg i løpet av 10 sekunder.
- Spørreskjema
- Passiv lytting
- Gir instrukser
- Gjennomfører trykking fra 0 til 2kg i løpet av 10 sekunder
- Spørreskjema

Instrukser for passiv lytting:

- Sitt, lytt og slapp av til musikken

Instrukser for aktiv lytting:

- Sitt, lytt og tapp med høyre fot til musikken.

Spørreskjema:

Vil vi gi spørreskjema etter hver gjennomføring.

- Likte du musikken? Ja/Nei
- Ranger musikken på en skala fra 1-9. 1=Veldig Dårlig, 5=Nøytral, 9=Veldig Bra
- Har du hørt musikken før? Ja/Nei

# Utvalg

Forsøkspersoner rekrutteres via epost eller informasjon på forelesninger til studenter ved Det psykologiske fakultet ved Universitetet i Bergen, ved Norges Handelshøyskole, ved BI Handelshøyskole campus Bergen, Høgskolen på Vestlandet. Eksklusjonskriterier er nevrologiske eller psykiatriske lidelser, rusavhengighet, bruk av reseptbelagte legemidler (kvinner kan bruke p-piller eller andre prevensjonsmidler). Alle deltakere skal være friske (via selv-rapportering). Deltakerne vil instrueres i å ikke drikke alkohol og til å seponere eventuell smertestillende minst 24 timer før eksperimentet. De vil også være blindet for studiens hypotese. Alle deltakere vil motta skriftlig informasjon og signere samtykkeskjema.

# Datainnsamling

Data blir samlet inn ved bruk av dataprogrammet MatLab.

# Eksperimentelle betingelser Trykkalgometer

I de to siste dagene før forsøksdagen blir forsøkspersonene instruert til å holde seg unna alkohol og smertestillende legemidler. Deltaker blir utsatt for ubehag med 30 sekunders mellomrom når det benyttes trykkalgometer. I løpet av 5 gjennomganger vil det byttes mellom bruk av høyre og venstre arm. Mengde trykk som ble benyttet noteres. Etter dette vil forsøksperson fylle ut et spørreskjema. Spørreskjemaet utformes av forskningsgruppen, og registrerer subjektiv opplevelse av ubehag og av musikken som benyttes. Samme prosedyre benyttes både ved passiv og aktiv lytting.

# Statistisk analyse

Dataene vil analyseres med en enveis ANOVA, der den ene faktoren (musikk) har to nivå (Passiv lytting vs. aktiv lytting). Hver forsøksperson vil delta i alle betingelsene.

Styrkeberegning ble gjort med G*Power, versjon 3.1.7 [32]. Statistisk styrke (1-β) var satt til

.80 (β er sannsynligheten for å begår en type II feil), alfa/signifikansnivå var satt til .05. Antatt effekt ble satt til d=0.50 (moderat effekt) og korrelasjonen mellom repeterte målinger ble satt til r=.50. Basert på disse parametre trengs minimum 21 forsøkspersoner for å påvise signifikante hovedeffekter av aktiv lytting og grad av opplevd smerte og en signifikant interaksjonseffekt (Lytting x Smerteinduksjon).

# Prosjektorganisering og personell

Prosjektleder:

Stefan Kölsch

Prosjektmedarbeidere:

Ståle Pallesen Sebastian Jentschke Stavros Skouras

Olav Gunnarson Jevne Brokke Chris-Andre Karlsen

Jørgen Aloysius Haug

# Prosjektressurser/utstyr

I tillegg til prosjektledelse og veiledningsfunksjoner ved Det psykologiske fakultet, UiB, vil det benyttes Wagners Trykkalgometer Force One.

# Planlagte arbeider:

“Reducing pain thresholds using a focused music listening technique”. Papiret vil skrives opp av tre bachelorstudenter under veiledning fra prosjektleder/veileder og biveileder.

Prosjektet vil også gjennomføres ved hjelp av fire emnestudenter.

# Kostnader

200kr i kompensasjon til alle deltagere.

# Finansieringsplan

Finansieres via midler fra prosjektleders forskningsbudsjett.

# Tidsplan

Avhengig av eventuell godkjenning fra Regional komité for medisinsk og helsefaglig forskningsetikk.

# Publisering

Ønskes å publiseres i vitenskapelig «Open Access» Tidsskrift.

# Etikk

Søknad om godkjenning fra Regional komité for medisinsk og helsefaglig forskningsetikk (REK) er under utarbeidelse.

Error in tag replacement: More than one holds the role ORGADV on the target: Error in tag replacement: More than one holds the role ORGADV on the target:

Stefan Kölsch

**7236 Reducing pain thresholds using a focused music listening technique Forskningsansvarlig**: Universitetet i Bergen

**Søker**: Stefan Kölsch

# REKs vurdering

Vi viser til søknad om prosjektendring for ovennevnte forskningsprosjekt mottatt 23.11.2020. Søknaden er behandlet av sekretariatet i REK vest på delegert fullmakt fra komiteen, med hjemmel i forskningsetikkforskriften § 7, første ledd, tredje punktum. Søknaden er vurdert med hjemmel i helseforskningsloven § 11.

*Ønsket endring:*

Prosjektleder ønsker å legge til en ny prosjektmedarbeider, Student Lucy Madeleine Werner.

*Vurdering:*

REK vest har ingen innvendinger mot ønsket endring.

# Vedtak

Godkjent

REK vest godkjenner prosjektendringen i samsvar med forelagt søknad, med hjemmel i helseforskningsloven § 11.

Med vennlig hilsen Fredrik Rongved rådgiver

# Klageadgang

Du kan klage på komiteens vedtak, jf. forvaltningsloven § 28 flg. Klagen sendes til REK vest. Klagefristen er tre uker fra du mottar dette brevet. Dersom vedtaket opprettholdes av REK vest, sendes klagen videre til Den nasjonale forskningsetiske komité for medisin og helsefag (NEM) for endelig vurdering.
