# Supplementary material for: Sensorimotor synchronization to music reduces pain
Source: PLoS One. 2023 Jul 28;18(7):e0289302. doi: 10.1371/journal.pone.0289302 (PMC10381080; doi:10.1371/journal.pone.0289302)
Supplement: S2 Protocol — (DOCX) [file pone.0289302.s002.docx]

**Research protocol: Reducing Pain Thresholds Using a Focused Music Listening**

**General information**

- **Protocol title:** Reducing Pain Thresholds Using a Focused Music Listening Technique
- **Protocol identifying number and date:** The study was registered as a clinical trial at ClinicalTrials.gov (registration number NCT05267795), and the trial was first posted on 04/03/2022
- **Name and address of the sponsor/funder:** University of Bergen, Bergen, Bergen Municipality, Norway, 5020
- **Name and title of the investigator(s) who is (are) responsible for conducting the research, and the address and telephone number(s) of the research site(s), including responsibilities of each:**
  - Univ.-Prof. Dr. Stefan Koelsch
  - Dept. of Biol. and Med. Psychology, University of Bergen, Postboks 7807, 5020 Bergen, Norway
  - Phone: +47 477 03 900
  - email: [stefan.koelsch@uib.no](mailto:stefan.koelsch@uib.no)
- **Name(s) and address(es) of the clinical laboratory(ies) and other medical and/or technical department(s) and/or institutions involved in the research:** None

**Project summary**

Pain-reducing effects of music listening are well-established, but the effects are small and their clinical relevance questionable. Recent theoretical advances, however, have proposed that synchronizing to music, such as clapping, tapping or dancing, has evolutionarily important social effects that are associated with activation of the endogenous opioid system (EOS; which supports both analgesia and social bonding). Thus, active sensorimotor synchronization to music could have stronger analgesic effects than simply listening to music. However, to the best of the investigators’ knowledge, the hypothesis of an EOS activation by sensorimotor synchronization to music has never been investigated. Accordingly, the investigators set up a test with the premise that if sensorimotor synchronization to music indeed activates the EOS, then it should have larger pain-reducing effects than simply listening to music. Using pressure algometry to the fingernails, specific amounts of pain were delivered to healthy adults either during music listening or silence, while either performing an active tapping task or a passive control task. As the dependent variable, perceived pain was rated on a scale ranging from 1 to 9 (1 = very little, 5 = medium, 9 =very strong). In addition, to pain ratings, participants provided ratings of their emotional state in terms of pleasantness as well as arousal, and then rated their familiarity with the music (also on scales ranging from 1 to 9). Emotion ratings were obtained to explore whether the mechanisms driving pain-reducing effects of sensorimotor synchronization to music include emotion. Each trial lasted about one minute (thus, the experiment had a duration of approximately 40 minutes). At the end of the experiment, participants also rated their preference for the music on a scale ranging from 1 to 9 (1 = *strongly disliked*, 5 = *medium*, 9 = *strongly liked*). Familiarity and preference ratings were obtained to elucidate possible contributions of these factors on pain reduction.

**Rationale & background information**

Pain-reducing effects of music listening are among the most widely studied and most replicable effects of music in clinical settings^1,2^. For example, a recent meta-analysis by Kuhlmann et al.^2^, with 7385 patients in 92 randomized controlled trials, reported that music listening interventions significantly reduce pain in surgical patients. However, across four different meta-analyses, the pain-reducing effects of music listening had only small to moderate effect sizes (and were inconsistent among the studies analyzed), which calls the clinical relevance of music for pain relief into question^1-4^. Notably, almost all of the studies included in the aforementioned meta-analyses used passive listening interventions, and to the best the investigators’ knowledge no study has compared pain-reducing effects between passive music listening and active engagement in music. The lack of research comparing pain-reducing effects between these two forms of music listening (i.e active vs. passive) is an important shortcoming because recent advances have proposed that active sensorimotor synchronization to music is associated with the activation of the endogenous opioid system (EOS), including the release of endorphins^5,6^. Notably, EOS activation supports both analgesia and social bonding^6^. Hence, recent evolutionary accounts have posited that the human capacity to synchronize movements to a musical pulse in a group (such as group singing, clapping, drumming, dancing) is an evolutionarily adaption because it stimulates social bonding^5-9^. However, to the best of the investigators’ knowledge, the hypothesis of an EOS activation by sensorimotor synchronization to music has never been investigated.

**Study goals and objectives**

The expected benefit of the study is that tapping to music, in addition to simply listening to music, may enhance pain-reducing effects both in a clinical context and in everyday life.

**Study design**

The study used a 2x2 within-subjects design. A naive random sample of 59 participants was included in the data analysis. Exclusion criteria were use of any prescription drugs, psychiatric or neurological disease, hearing impairment, and history of substance dependence (according to self-report). Participants should not consume alcohol nor any medicine for the treatment of pain at least 24 hours prior to the experiment. None of the participants had musical anhedonia according to the Barcelona Music Reward^10^. The duration of the experimental task was 40 min and the duration of the whole experiment approximately one hour. Data collection took place from 28.09.2020 to 30.10.2020.

**Methodology**

A graphic outline of the study design and procedures is provided in figure 1.

**Stimuli.** Music stimuli were presented with an average sound pressure of approximately 60 dB SPL over Beyerdynamic DT 770-PRO 250 Ohm headphones. 10 instrumental music experts were selected, each 30 seconds long. Each excerpt was played twice during the experimental task, once during the active task and once during the passive task. Stimuli were delivered using the Matlab-based toolbox Cogent 2000 (version 1.33).

**Pressure Algometry.** Pain was applied by the experimenter using a Wagner Force One Pressure Algometer (Wagner Instruments, Greenwich, USA). The equipment head (used to apply the pressure) had a diameter of 12mm and a surface area of 113mm^2^. Pain stimuli were delivered to index, middle and ring fingers of both hands. For each participant, pain thresholds were determined separately for each of the six fingers used for pain delivery, prior to the actual experiment. The average value of these pain thresholds was then computed, and for the actual experiment, only 50% of the participant’s average pain threshold was delivered as pain stimulus in each of the 40 experimental trials (e.g., if a participant had an average pain threshold of 5 kg, then a pressure stimulus of 2.5 kg was applied in the actual experiment). During the entire experiment, the applied pressure was measured with a 10 Hz sampling rate using MESURgauge Plus by Mark-10 (version 2.0.5).

**Procedure**. 40 experimental trials were delivered, 10 for each experimental trial type (Music Active, Music Passive, Silence Active, Silence Passive). The experimenter was blinded during the entire experimental procedure. Each trial started with an instruction screen where participants were either instructed to tap their right foot like a metronome (active tapping task) or to relax (passive control task). Then, participants pressed the space bar and either music started to play, or a silent period started, and participants either had to tap or relax (depending on the trial type), while looking at a fixation cross in the middle of the screen. After 20 seconds the experimenter applied pressure with the Algometer to a fingernail for 10 seconds. Then (after the pain application had stopped), participants indicated the pain they felt at the end of the pain application on a 9-point scale (1 = *very little*, 5 = *medium*, 9 = *very strong*), followed by a pleasantness rating (1 = *very uncomfortable*, 5 = *medium*, 9 = *very comfortable*) and an arousal rating (1 = *very calm*, 5 = *medium*, 9 = *very activated*). In music trials, participants also rated to which extent they were familiar with the music (1 = *not at all*, 5 = *partially known*, 9 = *well known*). Participants were aware that the experimenter could not see their ratings (so that they would not feel influenced in any way). Each trial lasted about one minute (thus, the experiment had a duration of approximately 40 minutes). After completing all 40 experimental trials, participants listened to the 10 music excerpts again and indicated their preference for each excerpt on a 9-point scale (1 = *strongly disliked*, 5 = *medium*, 9 = *strongly liked*).

**Figure 1**

Pain stimuli were delivered to index, middle and ring fingers of both hands. For each participant, pain thresholds were determined separately for each of the six fingers used for pain delivery, prior to the experimental task.

The experiment used a 2x2 design with the within-subject factors Condition (music, silence) and Task (active, passive), resulting in four experimental trial types: (a) Music Active (music with tapping); (b) Music Passive (music without tapping); (c) Silence Active (silence with tapping); and (d) Silence Passive (silence without tapping). Conditions were tested within subjects and the order of the conditions was counterbalanced. Specific pain levels were applied on the participants’ fingernails in each of 40 experimental trials using pressure algometry. At the end of each trial (after the presentation of a music excerpt or after a silent period), participants rated (1) their perceived pain, (2) their emotional state with regard to felt pleasantness and (3) felt arousal, as well as (4) their familiarity with the music excerpt (only during trials with music). All ratings were provided on a scale ranging from 1 to 9. The duration of the experimental task was approximately 40 minutes.

After the 40 experimental trials, participants provided preference ratings for each musical excerpt (also using a scale ranging from 1 to 9).

The duration of the whole experiment was approximately 60 minutes.

Experimental task

Preference rating

Find individual pain threshold

Collection of demographic data and written informed consent

**Safety considerations**

The safety of the participants was ensured throughout the experiment. The experimenter was present the entire time. Although participants felt pain during the experiment, the pain was not expected to be severe or long lasting. Participants could feel a slight pressure pain on the finger after pressing, but it was not expected to be prolonged. Participants could opt out of the experiment at any time.

**Follow-up**

No follow-up is foreseen for the research participants.

**Data management and statistical analysis**

The study was a quantitative experimental study. A naive random sample of 59 participants was included in the data analysis. The sample was a random sample. An a-priori sample size estimation using G*Power (version 3.1.9.6) indicated that for the detection of a small to medium effect size (*d* = 0.4) a sample of *n* = 59 is required with an α-level of 0.05 and a statistical power of 90% (one-tailed). The experiment was conducted on a Windows 10 Medion Laptop and the participants responded over the integrated keyboard on which the number keys 1 to 9 were marked in blue cause those keys and the space bar were used for the experimental task. Music stimuli were presented with an average sound pressure of approximately 60 dB SPL over Beyerdynamic DT 770-PRO 250 Ohm headphones. Pain was applied by the experimenter with a Wagner Force One Pressure Algometer (Wagner Instruments, Greenwich, USA). The equipment head (used to apply the pressure) had a diameter of 12mm and a surface area of 113mm2. Data was collected using the Matlab-based toolbox Cogent 2000 (version 1.33) and MESURgauge Plus by Mark-10 (version 2.0.5). Only the participant and the experimenter were present during the experimental procedure and the experimenter was blinded during the entire experimental procedure. Data collection took place from 28.09.2020 to 30.10.2020. Overall, a naive random sample of 62 participants took part in this study. Three participants were excluded from all analyses because of inadequate datasets due to technical difficulties. Thus, the sample for the main analysis consisted of 59 participants. We conducted LME analyses on single trial level, as this analysis allowed to reduce interindividual variance introduced by participants and music excerpts and decrease the risk for false positives^11^. For the second LME analysis two participants had a Cook’s Distance value above the cutoff (4/n, with *n* = 59) identifying them as influential data points for this model. Therefore, they were excluded only from the second LME analysis, which was thus conducted with a sample of 57 participants. No participants dropped out/declined participation. Conditions were tested within subjects and the order of the conditions was counterbalanced. Data analysis was computed using R (version 4.0.4). Data supporting the findings of this study and the R scripts used for the analyses, statistics and visualizations of this study will be provided by the senior author upon reasonable request.

**Quality assurance**

/

**Expected outcomes of the study**

A positive outcome of the present investigation may have wide-ranging implications for the use of music in clinical settings (as an adjunct treatment of pain)^12^, in music therapy (e.g. for chronic pain)^13^, and in everyday life (for the management of acute pain). Furthermore, it may shed new light on the mechanisms underlying pain reduction with music.

**Duration of the project**

Project start was at the 22.04.2019. The study and experimental procedure were carefully planned and approved by the Regional Committee for Medical and Health Research Ethics for Western Norway (Reference Number: 2019/1031) until the data collection started. Due to the Covid-19 pandemic, data collection could only start later than originally anticipated. Data collection took place from 28.09.2020 to 30.10.2020. Data analysis and writing the manuscript took place until the 23.10.202, where the manuscript was first submitted.

**Problems anticipated**

There were no difficulties anticipated.

**Project management**

L.W conducted the experiment, analyzed the results and wrote the original draft. S.S conceived the experiment, analyzed the results and reviewed the original draft. L.B analyzed the results and reviewed the original draft. S.P contributed to the methodology and reviewed the original draft. S.K. conceived the experiment, provided resources and supervision and essentially reviewed and edited the original draft.

**Ethics**

All participants provided written informed consent before enrollment and received a monetary compensation of 200 NOK following participation in the experiment.

The study was carried out in accordance with the guidelines of the Declaration of Helsinki and approved by the Regional Committee for Medical and Health Research Ethics for Western Norway (Reference Number: 2019/1031).

**References**

1. Cepeda, M. S., Carr, D. B., Lau, J. & Alvarez, H. Music for pain relief. *Cochrane Database Syst. Rev* **2**, (2006).
2. Kuhlmann, A. Y. R. *et al.* Meta-analysis evaluating music interventions for anxiety and pain in surgery. *Br J Surg* **105**, 773-783, doi:10.1002/bjs.10853 (2018).
3. Lee, J. H. The effects of music on pain: a meta-analysis. *Journal of music therapy* **53**, 430-477 (2016).
4. Martin-Saavedra, J. S., Vergara-Mendez, L. D., Pradilla, I., Velez-van-Meerbeke, A. & Talero-Gutierrez, C. Standardizing music characteristics for the management of pain: A systematic review and meta-analysis of clinical trials. *Complement Ther Med* **41**, 81-89, doi:10.1016/j.ctim.2018.07.008 (2018).
5. Granot, R. in *The Routledge Companion to Music Cognition* 101-111 (Routledge, 2017).
6. Tarr, B., Launay, J. & Dunbar, R. I. Music and social bonding:“self-other” merging and neurohormonal mechanisms. *Front. Psychol.* **5**, 1096 (2014).
7. Launay, J., Tarr, B. & Dunbar, R. I. M. Synchrony as an Adaptive Mechanism for Large-Scale Human Social Bonding. *Ethology* **122**, 779-789, doi:10.1111/eth.12528 (2016).
8. Savage, P. E. *et al.* Music as a coevolved system for social bonding. *Behavioral and Brain Sciences*, 1-36 (2020).
9. Tarr, B., Launay, J. & Dunbar, R. I. Silent disco: dancing in synchrony leads to elevated pain thresholds and social closeness. *Evol Hum Behav* **37**, 343-349, doi:10.1016/j.evolhumbehav.2016.02.004 (2016).
10. Mas-Herrero, E., Marco-Pallares, J., Lorenzo-Seva, U., Zatorre, R. J. & Rodriguez-Fornells, A. Individual differences in music reward experiences. *Music Percept.* **31**, 118-138 (2012).
11. Yarkoni, T. The generalizability crisis. *Behav. Brain Sci.*, 1-37 (2020).
12. Nilsson, U. The anxiety- and pain-reducing effects of music interventions: a systematic review. *AORN J* **87**, 780-807, doi:10.1016/j.aorn.2007.09.013 (2008).
13. Bradt, J. *et al.* The impact of music therapy versus music medicine on psychological outcomes and pain in cancer patients: a mixed methods study. *Support Care Cancer* **23**, 1261-1271, doi:10.1007/s00520-014-2478-7 (2015).
