# Supplementary material for: Sensorimotor synchronization to music reduces pain
Source: PLoS One. 2023 Jul 28;18(7):e0289302. doi: 10.1371/journal.pone.0289302 (PMC10381080; doi:10.1371/journal.pone.0289302)
Supplement: S3 Protocol — (DOCX) [file pone.0289302.s003.docx]

**Research Protocol**

# Project title

Reducing pain thresholds using a focused music listening technique

# Background

One of the most established findings in the field of music use in medicine is the reduction of pain when using music. Meta-analyses, on the other hand, suggest that the pain-reduction effect of music is small, despite its high reliability. The clinical relevance of the music is therefore not well established.

# Research question

However, previous studies that have investigated the pain reduction effect that music can cause have only used paradigms in passive listening to music. Techniques where a more active listening technique is used probably have a greater pain reduction effect. This project will investigate this problem by comparing the pain reduction effect of passive listening and active listening.

# Purpose

A positive outcome can have a significant impact on how the use of music during medical procedures can relieve pain and reduce psychological symptoms related to pain, such as anxiety and worry.

# METHODS

**Design**

The study is an experimental repeated within-subject measurement design which involves the subjects being subjected to discomfort using Wagner's pressure algometer Force One.

Plan Passive-Active

- Passive listening first
- Marks the finger to be pressed
- Gives instructions
- Performs printing from 0 to 2kg within 10 seconds
- Questionnaire
- Active listening
- Gives instructions
- Performs printing from 0 to 2kg within 10 seconds
- Questionnaire

Plan Active-Passive

- Active listening first
- Marks the finger to be pressed
- Gives instructions
- Performs printing from 0 to 2kg within 10 seconds
- Questionnaire
- Passive listening
- Gives instructions
- Performs printing from 0 to 2kg within 10 seconds
- Questionnaire

Instructions for passive listening:

- Sit, listen and relax to the music

Instructions for active listening:

- Sit, listen and tap your right foot to the music.

Questionnaire:

We will provide a questionnaire after each implementation.

- - Did you like the music? Yes No
  - Rate the music on a scale from 1-9. 1=Very Bad, 5=Neutral, 9=Very Good
  - Have you heard the music before? Yes No

# Selection

Subjects are recruited via e-mail or information at lectures to students at the Faculty of Psychology at the University of Bergen, at the Norwegian School of Business, at the BI School of Business Bergen campus, the College of Western Norway. Exclusion criteria are neurological or psychiatric disorders, drug addiction, use of prescription drugs (women can use birth control pills or other contraceptives). All participants must be healthy (via self-reporting). Participants will be instructed not to drink alcohol and to discontinue any painkillers at least 24 hours before the experiment. They will also be blinded to the study's hypothesis. All participants will receive written information and sign a consent form.

**Data collection**

Data is collected using the computer program MatLab.

# Experimental conditions

**Pressure algometry**

In the last two days before the day of the experiment, the subjects are instructed to stay away from alcohol and painkillers. The participant is exposed to discomfort at 30 second intervals when the pressure algometer is used. During 5 reviews, it will be switched between the use of the right and left arm. The amount of pressure that was used is noted. After this, the subject will fill in a questionnaire. The questionnaire is designed by the research group, and records the subjective experience of discomfort and of the music used. The same procedure is used for both passive and active listening.

**Statistical analysis**

The data will be analyzed with a one-way ANOVA, where one factor (music) has two levels (Passive listening vs. active listening). Each subject will participate in all conditions.

# Power calculation was done with G*Power, version 3.1.7 [32]. Statistical power (1-β) was set .80 (β is the probability of committing a type II error), alpha/significance level was set at .05. Assumed effect was set to d=0.50 (moderate effect) and the correlation between repeated measurements was set to r=.50. Based on these parameters, a minimum of 21 subjects are needed to demonstrate significant main effects of active listening and degree of perceived pain and a significant interaction effect (Listening x Pain induction).

**Project organization and personnel**

Project Manager:

Stefan Kölsch

Project staff:

Ståle Pallesen

Sebastian Jentschke

Stavros Skouras

Olav Gunnarson

Jevne Brokke

Chris-Andre Karlsen

Jørgen Aloysius Haug

Laura Bechtold

Lucy Madeleine Werner

**Project resources/equipment**

In addition to project management and guidance functions at the Faculty of Psychology, UiB, Wagner's Trykkalgometer Force One will be used

**Planned works:**

"Reducing pain thresholds using a focused music listening technique". The paper will be written by three bachelor students under the guidance of the project manager/supervisor and co-supervisor.

The project will also be carried out with the help of four course students.

**Costs**

NOK 200 in compensation for all participants.

**Financing plan**

Funded via funds from the project manager's research budget.

**Schedule**

Subject to possible approval from the Regional Committee for Medical and Healthcare Research Ethics.

**Publication**

Would like to publish in a scientific "Open Access" journal.

**Ethics**

An application for approval from the Regional Committee for Medical and Health Research Ethics (REK) is being prepared.

Error in tag replacement: More than one holds the role ORGADV on the target: Error in tag replacement: More than one holds the role ORGADV on the target:

Stefan Kölsch

**7236 Reducing pain thresholds using a focused music listening technique
Research manager**: Universitetet i Bergen

**Applicant:** Stefan Kölsch

**REK's assessment**

We refer to the application for project change for the above-mentioned research project received on 23 November 2020. The application has been processed by the secretariat of REK west on delegated authority from the committee, based on the research ethics regulations § 7, first paragraph, third sentence. The application has been assessed on the basis of section 11 of the Norwegian Health Research Act.

*Desired change:*

The project manager wants to add a new project employee, Student Lucy Madeleine Werner

*Rating:*

REK west has no objections to the requested change.

**Resolution**

Approved

REK west approves the project change in accordance with the submitted application, based on section 11 of the Norwegian Health Research Act.

Sincerely,

Fredrik Rongved

Adviser

**Access to appeal**

You can appeal against the committee's decision, cf. Norwegian Public Administration Act § 28 et seq. The appeal is sent to REK vest. The appeal deadline is three weeks from when you receive this letter. If the decision is upheld by REK West, the complaint is forwarded to the National Research Ethics Committee for Medicine and Health Sciences (NEM) for final assessment.
