# Supplementary material for: Sensorimotor synchronization to music reduces pain
Source: PLoS One. 2023 Jul 28;18(7):e0289302. doi: 10.1371/journal.pone.0289302 (PMC10381080; doi:10.1371/journal.pone.0289302)
Supplement: S1 Table — (DOCX) [file pone.0289302.s005.docx]

**S1 Table**

| Composer | Title | Beats per minute | | Genre |
| --- | --- | --- | --- | --- |
| Aqua | Barbie Girl | | 140 | Eurodance, Electropop |
| Bon Jovi | Living on a Prayer | | 122 | (Arena) Rock |
| Bruno Mars | The Lazy Song | | 88 | Pop |
| Charlie Puth | Marvin Gaye | | 110 | Pop |
| Glee/Michael Jackson | Smooth Criminal | | 135 | Pop |
| Mann | Buzzin | | 104 | Hip-Hop/Rap |
| MGMT | Kids | | 123 | Electro, Pop, Alternative/Indie |
| Selena Gomez | Love You Like A Love Song | | 117 | Electropop |
| The XX | Intro | | 100 | Trip hop |
| Toto | Africa | | 93 | (Soft) rock |

*Music excerpts played during the experiment*

*Note.* Each music excerpt was 30 seconds long and was played once for each task (active, passive).
