# Supplementary material for: Sensorimotor synchronization to music reduces pain
Source: PLoS One. 2023 Jul 28;18(7):e0289302. doi: 10.1371/journal.pone.0289302 (PMC10381080; doi:10.1371/journal.pone.0289302)
Supplement: S2 Table — (DOCX) [file pone.0289302.s006.docx]

|  | Pressure (normalized) |  | Duration (in seconds) |
| --- | --- | --- | --- |
|  | *M (SD)* |  | *M (SD)* |
| Music Active | 0.84 (0.05) |  | 9.98 (0.13) |
| Music Passive | 0.84 (0.05) |  | 10.00 (0.00) |
| Silence Active | 0.84 (0.05) |  | 9.95 (0.22) |
| Silence Passive | 0.83 (0.05) |  | 9.97 (0.26) |

**S2 Table**

*Descriptive statistics of the applied pressure and duration of the applied pressure*

*Note.* For both measures the mean (*M*) and the standard deviation (*SD*) are displayed. The applied pressure was normalized in relation to the respective individual 50% mean pain threshold value of each participant and the duration of the applied pressure was measured in seconds.
