# Supplementary material for: Sensorimotor synchronization to music reduces pain
Source: PLoS One. 2023 Jul 28;18(7):e0289302. doi: 10.1371/journal.pone.0289302 (PMC10381080; doi:10.1371/journal.pone.0289302)
Supplement: S3 Table — (DOCX) [file pone.0289302.s007.docx]

**S3 Table**

*Inferential Statistics of the LME Analysis on single trial perceived pain*

| *Predictor* | *β* | *SE* | *df* | *F* | *t* | *p* |
| --- | --- | --- | --- | --- | --- | --- |
| Condition | -0.72 | 0.20 | 3.45 | 13.78 | -3.71 | .027* |
| Task | -0.20 | 0.07 | 2289.00 | 8.01 | -2.83 | .005** |
| Condition x Task | -0.93 | -7.17 | 2289.00 | 1.24 | -1.12 | .265 |

*Note*. LME = linear mixed effects, *SE* = standard error. The sign of the beta estimates shows the direction of main effects of *Condition* (music [+0.5] vs. silence [-0.5]) and *Task* (active [+0.5] vs. passive [-0.5]).

* indicates *p* < .05, ** indicates *p* < .01
