# Supplementary material for: Sensorimotor synchronization to music reduces pain
Source: PLoS One. 2023 Jul 28;18(7):e0289302. doi: 10.1371/journal.pone.0289302 (PMC10381080; doi:10.1371/journal.pone.0289302)
Supplement: S4 Table — (DOCX) [file pone.0289302.s008.docx]

**S4 Table**

| Comparison | *Mean (SE)* | *t (df)* | *p* | *Cohen’s d* | |
| --- | --- | --- | --- | --- | --- |
| Music Active vs. Music Passive | -0.28 (0.08) | -3.50 (58) | <.001** | | 0.46 |
| Music Active vs. Silence Active | -0.81 (0.12) | -6.97 (58) | <.001** | | 0.91 |
| Music Active vs. Silence Passive | -0.93 (0.13) | -7.17 (58) | <.001** | | 0.93 |
| Music Passive vs. Silence Active | -0.52 (0.14) | -3.77 (58) | <.001** | | 0.49 |
| Music Passive vs. Silence Passive | -0.64 (0.15) | -4.38 (58) | <.001** | | 0.57 |
| Silence Active vs. Silence Passive | -0.12 (0.09) | -1.31 (58) | .098 | | 0.17 |

*Paired-Samples t-tests (one-tailed) on the perceived pain between all four experimental conditions*

*Note.* The mean difference (*Mean*) and the standard error (*SE*) of each comparison as well as the results of each paired-samples t-test are displayed (*df* = degrees of freedom). One-sided p-values are reported. All comparisons were highly significant except the comparison of silence with tapping (Silence Active) and silence without tapping (Silence Passive).

** indicates *p* < .01.
