# Supplementary material for: Sensorimotor synchronization to music reduces pain
Source: PLoS One. 2023 Jul 28;18(7):e0289302. doi: 10.1371/journal.pone.0289302 (PMC10381080; doi:10.1371/journal.pone.0289302)
Supplement: S5 Table — (DOCX) [file pone.0289302.s009.docx]

**S5 Table**

*Inferential Statistics of the LME Analysis on the single trial perceived pain for music trials*

| *Predictor* | *β* | *SE* | *df* | *t* | *F* | *p* |
| --- | --- | --- | --- | --- | --- | --- |
| Task | -0.28 | 0.10 | 1070.35 | -2.77 | 7.64 | .006** |
| Preference | -0.14 | 0.03 | 814.27 | -4.47 | 19.98 | <.001*** |
| Familiarity | 0.03 | 0.02 | 387.50 | 1.23 | 1.50 | .221 |
| Task x Preference | -0.04 | 0.05 | 1070.72 | -0.75 | 0.56 | .453 |

*Note*. LME = linear mixed effects, *SE* = standard error. The sign of the beta estimates shows the direction of main effects of *Task* (active [+0.5] vs. passive [-0.5]) and *Preference* (rated on a scale ranging from 1 to 9 and mean-centered) and of the covariate *Familiarity* (scale ranging from 1 to 9).

**indicates *p* < .01, *** indicates *p* < .001
