# Supplementary material for: Sensorimotor synchronization to music reduces pain
Source: PLoS One. 2023 Jul 28;18(7):e0289302. doi: 10.1371/journal.pone.0289302 (PMC10381080; doi:10.1371/journal.pone.0289302)
Supplement: S6 Table — (DOCX) [file pone.0289302.s010.docx]

**S6 Table**

*Inferential Statistics of the LME Analysis on the single trial felt pleasantness*

| *Predictor* | *β* | *SE* | *df* | *t* | *F* | *p* |
| --- | --- | --- | --- | --- | --- | --- |
| Condition | 1.02 | 0.25 | 5.19 | 4.03 | 16.20 | .009** |
| Task | 0.06 | 0.07 | 2289.00 | 0.86 | 0.74 | .391 |
| Condition x Task | 0.24 | 0.13 | 2289.00 | 1.79 | 3.21 | .073 |

*Note*. LME = linear mixed effects, *SE* = standard error. The sign of the beta estimates shows the direction of main effects of *Condition* (music [+0.5] vs. silence [-0.5]) and *Task* (active [+0.5] vs. passive [-0.5]).

** indicates *p* < .01
