# Supplementary material for: Sensorimotor synchronization to music reduces pain
Source: PLoS One. 2023 Jul 28;18(7):e0289302. doi: 10.1371/journal.pone.0289302 (PMC10381080; doi:10.1371/journal.pone.0289302)
Supplement: S7 Table — (DOCX) [file pone.0289302.s011.docx]

**S7 Table**

*Descriptive statistics of the felt arousal per experimental condition*

|  | *M (SD)* |
| --- | --- |
| Music Active | 4.74 (1.27) |
| Music Passive | 4.37 (1.28) |
| Silence Active | 5.16 (1.42) |
| Silence Passive | 4.75 (1.41) |

*Note.* The mean (*M*) and the standard deviation (*SD*) of the felt arousal (rated on a scale ranging from 1 to 9) are displayed. Felt arousal was highest for silence with tapping (Silence Active) and lowest for music without tapping (Music Passive). There was almost no difference between music with tapping (Music Active) and silence without tapping (Silence Passive).
