# Supplementary material for: Sensorimotor synchronization to music reduces pain
Source: PLoS One. 2023 Jul 28;18(7):e0289302. doi: 10.1371/journal.pone.0289302 (PMC10381080; doi:10.1371/journal.pone.0289302)
Supplement: S1 File — (DOCX) [file pone.0289302.s012.docx]

**S1 Supporting Information. Supplementary methods for the pressure data.**

In order, to assure that there was no significant difference in the applied pressure and the duration of the applied pressure that could influence the main findings two separate 2x2 repeated measures ANOVAs (within-subjects) with the inner subject factors *Condition* (music, silence) and *Task* (active, passive) were performed with the R package rstatix (version 0.7.0) [1]. The dependent variables were the applied pressure normalized in relation to the respective individual 50% mean pain threshold value of each participant and the duration of the applied pressure in seconds. For significance, an α-level of 0.05 was assumed. Both 2x2 repeated measures ANOVAs showed no significant effects, all *p* > .209, indicating that the applied pressure and the duration of the applied pressure did not differ between the experimental conditions and therefore did not influence the main findings. Descriptive data of the applied pressure and the duration of the applied pressure are shown in S2 Table.

**References**

1. Kassambara A. Rstatix: pipe-friendly framework for basic statistical tests. R package version 070. 2020.
