# Supplementary material for: Sensorimotor synchronization to music reduces pain
Source: PLoS One. 2023 Jul 28;18(7):e0289302. doi: 10.1371/journal.pone.0289302 (PMC10381080; doi:10.1371/journal.pone.0289302)
Supplement: S2 File — (DOCX) [file pone.0289302.s013.docx]

**S2 Supporting Information. Experimental Instructions.**

“This experiment consists of fourty different rounds. You will be advised to look at the screen during each round. In each round you will receive a pressure on a fingernail (as just demonstrated by the experimenter). Between the rounds you will be asked to answer some questions. It is important that you answer as precisely as possible. You are supposed to answer on a scale ranging from 1-9. Please press the number on the keyboard that corresponds to your selection.

In some rounds you will be asked to just relax while music is playing. In other rounds you will be asked to focus on the music while tapping the tempo of the music with one foot (like a metronome). It is very important that you only tap your foot to the music in the rounds where you are asked to do so (and not in the rounds where you are asked to just relax).

In the experiment there will also be rounds without music. In some rounds without music you will be asked to tap with your foot like a metronome. In those rounds it is important that you do NOT think about music or hum a melody to make it easier to keep pace with your foot (even though that could make it easier to tap like a metronome).

Note that between rounds, the experimenter will not talk to you or look at you. This is done to prevent influencing the experiment. The experiment will last for about 40 minutes. In case you have any questions please ask the experimenter now. If you have no questions please press "space" to start the experiment.”
