# Supplementary material for: Sensorimotor synchronization to music reduces pain
Source: PLoS One. 2023 Jul 28;18(7):e0289302. doi: 10.1371/journal.pone.0289302 (PMC10381080; doi:10.1371/journal.pone.0289302)
Supplement: S3 File — (DOCX) [file pone.0289302.s014.docx]

**S3 Supporting Information. Supplementary methods: Experimental design and data analysis on emotional mechanisms underlying the pain-reducing effect of sensorimotor synchronization to music.**

We conducted two additional LME analyses to test our assumption that the mechanisms driving the pain-reducing effects of sensorimotor synchronization to music include emotion: (1) The first of these two analyses was performed on the single trial perceived pain but included music trials only. Furthermore, this model included the categorical fixed-effect factor *Task* (active [+0.5] and passive [-0.5]) and the mean-centered continuous fixed-effect factor *Preference* (rated on a scale ranging from 1 to 9) as well as their interaction as predictors. In addition, the model included the main effect of the mean-centered continuous fixed-effect factor *Familiarity* (scale ranging from 1 to 9) as a covariate to test for a potential confounding effect. (2) The second model to study emotional mechanisms was regarding the level of felt pleasantness on the single trial level. The model included the two categorical fixed-effects factors *Condition* (music [+0.5] and silence [-0.5]) and *Task* (active [+0.5] and passive [-0.5]), as well as their interaction as predictors for the level of felt pleasantness in each individual experimental trial (rated on a scale ranging from 1 to 9). The random-effects factors for the two LME analyses on the emotional mechanisms were the same as specified for the main LME analysis and we used the same R packages as for the main LME analysis (see main text for details). The investigation for statistical outliers on the participant level showed that for the first LME analysis two participants had a Cook’s Distance value above the outlier cutoff (4/*n*, with *n* = 59 in all analyses). Therefore, they were excluded only from this LME analysis, which was thus conducted with a sample of 57 participants. In the second LME analysis on emotional mechanisms, no outliers were detected, and the model was conducted with a sample of 59 participants.
