# Supplementary material for: Sensorimotor synchronization to music reduces pain
Source: PLoS One. 2023 Jul 28;18(7):e0289302. doi: 10.1371/journal.pone.0289302 (PMC10381080; doi:10.1371/journal.pone.0289302)
Supplement: S4 File — (DOCX) [file pone.0289302.s015.docx]

**S4 Supporting Information. Data analysis & results on the pain-reducing effect of sensorimotor synchronization to music including the mean-centered EHI as a covariate to test for confounding effects of handedness.**

We performed the same LME analysis as our main analysis, with the model this time additionally including the main effect of the mean-centered continuous fixed-effect factor *EHI* as a covariate to test for confounding effects of handedness (for details on the experimental design and data analysis see main text). No effect of the covariate *EHI* was observed (*β =* 0.33*, SE =* 0.35*, p* = .348) and the result pattern remained the same (see results section in the main text).
