# Supplementary material for: Sensorimotor synchronization to music reduces pain
Source: PLoS One. 2023 Jul 28;18(7):e0289302. doi: 10.1371/journal.pone.0289302 (PMC10381080; doi:10.1371/journal.pone.0289302)
Supplement: S5 File — (DOCX) [file pone.0289302.s016.docx]

**S5 Supporting Information. Data analysis & results on the pain-reducing effect of sensorimotor synchronization to music including gender as a covariate to test for confounding effects.**

We performed the same LME analysis as our main analysis, with the model this time additionally including the main effect of the categorical fixed-effect factor *Gende*r (male [+0.5] and female [-0.5]) as a covariate to test for confounding effects (for details on the experimental design and data analysis see main text). No effect of the covariate *Gender* was observed (*β =* 0.21*, SE =* 0.31*, p* = .504) and the result pattern remained the same (see results section in the main text).
